# Multi-method *in vitro* assessment of ultraviolet-C treatment against conidia and hyphal fragments of *Botrytis cinerea*

**DOI:** 10.1128/aem.01413-25

**Published:** 2025-11-24

**Authors:** Makayla Bellino, Joy Waite-Cusic, Qingyang Wang

**Affiliations:** 1 Department of Food Science and Technology, Oregon State University 2694, Corvallis, Oregon, USA; Anses, Maisons-Alfort Laboratory for Food Safety, Maisons-Alfort, France

**Keywords:** gray mold, UV-C, antifungal, fungal spores, fragmentation, spoilage control

## Abstract

*Botrytis cinerea* is a significant postharvest fungal pathogen responsible for substantial losses in a variety of fresh produce. Ultraviolet-C (UV-C) irradiation is a potential intervention that could reduce postharvest losses due to fungal disease. However, the diversity of fungal reproductive forms and lack of standardization in antifungal assessment methods complicate the evaluation of UV-C efficacy. This study evaluated the *in vitro* antifungal efficacy of UV-C against *B. cinerea* conidia and hyphal fragments (HF) treated in liquid suspension and on agar surfaces. *B. cinerea* viability was assessed using several methods: colony counts (from agar surface and suspension treatments), growth kinetics (suspension treatment), and conidial germination assays (suspension treatment). UV-C treatment caused significant, dose-dependent reductions in viability across all methods and for both reproductive forms. Surface treatments yielded the highest log reductions (>3.5 log reduction of conidia at 543.6 mJ/cm²), while suspension treatments showed comparatively modest reductions (~1–2 log reduction of conidia at 543.6 mJ/cm², depending on the method). Growth kinetics showed extended lag phases following UV-C exposure. Conidial germination was reduced by up to 95%, with diminishing efficacy at higher doses. While conidia generally exhibited greater sensitivity to UV-C than HF, the magnitude of inactivation varied by assay. A mixed-effects model confirmed that UV-C dose, reproductive form, and assessment method had a significant impact on fungal inactivation.

**IMPORTANCE:**

This study systematically compared UV-C effects on conidia and HF using a multi-method in vitro approach, underscoring the utility of UV-C for fungal control and providing critical insights for designing effective, context-aware antifungal strategies. The results have practical implications for improving postharvest sanitation protocols and minimizing produce spoilage in storage and packing environments.

## INTRODUCTION

*Botrytis cinerea*, the causal agent of Botrytis blight and gray mold rot, is a widespread fungal pathogen affecting hundreds of plant species, including many economically important crops (1). It contributes to major economic losses in both pre- and postharvest settings. Global economic losses caused by *B. cinerea* are substantial and difficult to quantify precisely; however, annual expenditures on control measures alone are estimated to exceed $1 billion (2). A key challenge in managing *B. cinerea* is its high capacity to mutate, which facilitates rapid adaptation and resistance to chemical sanitizers and fungicides (3, 4). For example, *B. cinerea* isolated from blueberries in California and Washington has shown reduced sensitivity to multiple commonly used fungicide classes (5), and similar trends were observed in grapes in Michigan between 2014 and 2018 (6). These resistance issues underscore the need for alternative or complementary approaches to control *B. cinerea*.

Ultraviolet-C (UV-C) light is a form of non-ionizing electromagnetic radiation (200 and 280 nm) and has long been recognized for its antimicrobial properties. The primary mechanism of antimicrobial activity is covalent crosslinking of nucleic acid residues which inhibits strand separation and thereby prevents transcription and replication (7). UV-C was approved as a food processing technology by the US Food and Drug Administration in 1977 and remains approved for use in food processing with specific limitations (8, 9). UV-C is a promising alternative to chemical fungicides for managing postharvest fungal pathogens. Most of the existing literature on the antimicrobial efficacy of UV-C has focused on bacterial inactivation, whereas most UV-C research on plant pathogens has been focused on hormesis. The direct fungicidal or fungistatic effects of UV-C on *B. cinerea* propagules remain relatively underexplored.

A complication in antifungal evaluations is the biological diversity of fungal reproductive strategies and their potential differences in sensitivity to antifungal treatments. *B. cinerea* can reproduce via conidia (asexual reproductive spores) or vegetative hyphal fragments (HF). Both cell types are capable of initiating infection (10–12). While conidia are typically the focus of *in vitro* antifungal assays, HFs, produced through mycelial fragmentation, are also viable and infectious fungal propagules. In postharvest environments such as storage and packing facilities, HF being a source of infection is particularly relevant, where mechanical handling could fragment mycelia existing on diseased produce (13). These fragments can then spread to nearby produce and facilitate host-to-host infection. The spread of hyphae-mediated disease has been a noted problem particularly during extended storage holding periods even at cold refrigeration temperature. Sbodio et al. (14) demonstrated how adjacently infected fruits (apples, oranges, and tomatoes) can spread disease to healthy unwounded fruits over storage through hyphal colonization (14). Despite their relevance, HFs are often overlooked in antifungal studies, which may lead to incomplete or underestimated assessments of fungal control strategies.

There is a diversity of antifungal assessment methods reported in the literature and a lack of a unified or standardized methodology confounds the interpretation of UV-C efficacy between studies. *In vitro* studies commonly use methods such as conidial germination rates (5, 15–17), yeast and mold colony counts (18, 19), or mycelial growth measurements (20–23), each capturing different aspects of fungal response. Results from each method are not always comparable and may yield inconsistent results. A multi-method approach is therefore critical to comprehensively characterize treatment effects and inform practical applications.

The objectives of this study were to (i) evaluate the efficacy of UV-C treatment against different fungal propagule types of *B. cinerea*: conidia and HF, and (ii) compare commonly used antifungal assessment methods, including (i) colony counts after exposure on solid or in liquid matrices, (ii) colony growth kinetics, and (iii) conidial germination assays, for evaluating UV-C treatment performance. Findings from this research enhance the understanding of UV-C as a postharvest fungal control strategy and support the development of industrial applications aimed at reducing produce losses caused by *B. cinerea*.

## MATERIALS AND METHODS

### *B. cinerea* strain information and culturing procedures

*B. cinerea* BC01 was originally isolated from blueberries grown in Oregon and kindly provided by Virginia Stockwell at USDA-ARS Horticulture Crops Research Unit (Corvallis, OR, USA). This strain was received on Potato Dextrose Agar (PDA; pH 5.6; BD Difco, Becton, NJ) and subcultured onto fresh PDA plates. Cultures were incubated at 25°C for 7 days to encourage sporulation. Conidia were harvested by flooding each PDA plate with 10 mL of sterile deionized (DI) water, scraping the surface with a sterile cell spreader, and filtering the resulting suspension with sterile gauze (mesh size grade 10; VWR, Radnor, PA). The filtered conidia suspension was transferred to a cryogenic tube and mixed at a 1:1 ratio with 50% glycerol. The resulting suspension was stored at −80°C for long-term preservation. To revive culture, an aliquot (100 µL) of the conidia suspension was spread onto PDA and incubated at 25°C for 5–7 days. An agar plug (6 mm diameter) was excised from the actively growing colony and transferred to a new sterile PDA plate, which was incubated as described above. This culture was subsequently used to prepare either conidia or HF inocula as described below.

### Inoculum preparation: fungal conidia

Conidia suspensions were prepared by flooding 7- to 10-day-old *B. cinerea* cultures on a PDA plate with 10 mL of sterile DI water and gently scraping the colony with a cell spreader. Harvest timing of the cultures was adjusted based on the development of visible conidial pigmentation. The resulting cell suspension was filtered through sterile gauze (mesh size 10; VWR, Radnor, PA) to remove large hyphal pieces and create the conidia suspension. Suspensions were used immediately after preparation. Conidia density (expressed as CFU/mL) was determined using a hemocytometer and validated by plate counts. For plate counts, serial dilutions of the suspension were performed and spread-plated on PDA plates. Plates were inoculated at 25°C for 3 days before colony enumeration.

### Inoculum preparation: HFs

HF suspensions were prepared from 5 to 7-day-old *B. cinerea* cultures on PDA plates. Cultures were harvested when white, non-pigmented hyphal growth was visible at the colony margins, indicating an absence of sporulation. Hyphae were collected by scraping the entire surface of two culture plates with a sterile scalpel. The scraped mycelia from both plates were then transferred to 8–10 mL of DI water and homogenized at 20,000 rpm for 5 min with a homogenizer (Fisher 850 Homogenizer, Pittsburgh, PA, USA). The resulting HF density (expressed as CFU/mL) was assessed by plate counts as described above. Due to the irregular shape and size of the HF, they could not be accurately quantified using a hemocytometer; therefore, this method was excluded for HF enumeration.

### UV-C treatment and dose calculation

All UV-C treatments were performed using a Spectroline Microprocessor-Controlled XL1000 UV Crosslinker (Spectro-UV, Farmingdale, NY, USA), equipped with five 8W SW BLE-8T254 shortwave tubes (254 nm) arranged in parallel at the top of the inner chamber. UV-C dose (mJ/cm^2^) was calculated based on irradiance measured using a dosimeter (General UVC Digital Light Meter 220–275 nm, Worthington Industries, Inc. Columbus, OH, USA). Light intensity (µW/cm^2^ at 254 nm) was recorded at the geometric center of the exposure surface over 10 min, not including the first 300 s of chamber warm-up, and average irradiance intensity was calculated from three random observations (4529.67 ± 75.43 μW/cm^2^). The UV-C dose was calculated by multiplying the average intensity by the exposure time and converted to mJ/cm^2^. Treatments of 0, 30, 60, 90, or 120 s of UV-C exposure (corresponding to 0, 135.9, 271.8, 407.7, 543.6 ± 2.3 mJ/cm^2^) were conducted in three independent replicates, with each replicate performed on a different day using independently prepared inocula.

### Agar surface treatment viable CFU counts of conidia and HF

Conidia and HF suspensions (prepared as described in Sections 2.2 and 2.3) were serially diluted (10^0^–10^−3^) in sterile DI water, and each dilution was spread-plated (100 µL) onto PDA plates and allowed to dry (~5 min) in ambient conditions near an electric Bunsen burner. For UV-C treatment, a single inoculated PDA plate was placed in the geometric center of the UV Crosslinker, the Petri dish lid was removed, and the open plate was exposed for 0–120 s. Each treatment combination (dilution × UV-C dose) was performed in duplicate on each treatment day (*n* = 3). After treatment, lids were replaced, and the treated plates were incubated at 25°C for 48 h (HF) or 72 h (conidia) prior to the enumeration of colonies. Surviving populations were enumerated, and mean values were converted to log_10_ CFU/mL for visualization and statistical analysis; log reductions were calculated by subtracting the surviving log CFU/mL from controls (0 mJ/cm^2^). Values below the detection limit (1 log CFU/mL) were assigned a colony count of 1 for analysis. A mechanistic growth model was fit to the surviving cell density as a function of UV-C dose. A fixed effects mixed model and Tukey-Kramer honestly significant difference (HSD) *post hoc* tests were applied to assess the effect of UV-C dose and inoculum type on fungal inactivation.

### Liquid suspension treatment viable CFU counts of conidia and HF

Three independent conidia and HF suspensions were prepared as described above. From each suspension sample, an aliquot of 400 µL was transferred to a single well of a 12-well plate (Corning Costar Clear TC-treated, Glendale, AR, USA), yielding a liquid depth of approximately 1.2 mm. The inoculated well was then placed in the geometric center of the UV Crosslinker and treated for the appropriate time (0–120 s). After treatment, the sample was serially diluted and spread plated onto PDA plates. This process was repeated with additional samples using the remaining wells in the 12-well plate. All plates were incubated at 25°C, and colonies were enumerated after 48 h (HF) and 72 h (conidia). Plates were enumerated and converted to log CFU/mL; reduction was calculated by subtracting from the replicate control (0 mJ/cm^2^). A generalized linear mixed model followed by Tukey’s HSD *post hoc* tests assessed the effect of UV-C dose. A fixed effects mixed model and Tukey’s HSD *post hoc* tests were applied to assess the effect of UV-C dose and inoculum type on fungal inactivation.

### Fungal growth kinetics (OD) post UV-C treatment

Optical density (OD)-based measurement of fungal growth on solid media was adapted from the method described by Cánovas et al. (24). Conidia and HF suspensions were prepared and aliquoted (400 µL) into sterile 12-well plates (Corning Costar Clear TC-treated, Glendale, AR, USA) and treated with UV-C as described previously (section 2.6). After treatment, three aliquots (40 µL) per condition were transferred to individual wells of a sterile 96-well plate (Corning Costar Clear TC-treated, Glendale, AR, USA) containing 100 µL PDA. For each condition, three technical replicates were plated for conidia suspensions, and six technical replicates for HF suspensions. This setup was repeated using three independently prepared inocula (i.e., three biological replications). Each 96-well plate was incubated at ambient temperature in a SpectraMax M2 Microplate Reader (Molecular Devices, San Jose, CA, USA) and the OD at 595 nm (OD_595_) was measured every hour for 96 h. Blank PDA wells had an average baseline OD_595_ value of approximately 0.25. Initial OD_₅₉₅_ readings were higher for HF suspensions due to the brown hue that developed after homogenization. To normalize, the average *t*_0_ OD_₅₉₅_ difference between conidia and HF suspensions at each dose was subtracted from all HF wells. Resulting normalized data were exported and fitted using a four-parameter logistic (4P) non-linear regression model to build growth curves. Key parameters (25), including growth rate, time to inflection point (*T*_IP_), and delta lag phase duration (△LPD), were compared across doses and inoculum types using least squares analysis with Tukey’s *post hoc* testing. Changes in lag phase were also fit to an exponential decay model, and 95% confidence intervals of model parameters were used to compare inoculum responses and predict initial viable counts for treated samples.

#### Diluted growth curve samples

To establish a relationship between initial cell density and growth kinetics, a dilution series (10⁰ to 10⁻²) was prepared from a single biological replicate of each inoculum type (conidia and HF). Each dilution was dispensed into three wells of a 96-well microplate (Corning Costar Clear TC-treated, Glendale, AR, USA) containing 100 µL of PDA per well. The exact cell concentration of each dilution was determined via standard plate counts and expressed as log CFU/mL. Plates were incubated at ambient temperature within a SpectraMax M2 Microplate Reader (Molecular Devices, San Jose, CA, USA), and OD at 595 nm (OD_₅₉₅_) was measured at hourly intervals for 96 h. Growth curves were generated using a 4P non-linear regression model. The LPD, calculated from each fitted curve, was then correlated with the corresponding log CFU/mL using a three-parameter exponential decay (3P) model. This relationship was subsequently used to construct a predictive model to estimate viable cell densities in UV-C-treated samples based on their observed LPD values. Estimated log CFU/mL values were used to calculate log reductions by subtracting the predicted counts of treated samples from those of untreated controls (0 mJ/cm²).

### Conidial germination assessment

The method for conidial germination assessment was modified from Zhou et al. (17). An aliquot of 400 µL conidia suspensions prepared as described previously was transferred to a single well of a 12-well plate (Corning Costar Clear TC-treated, Glendale, AR, USA). An individual filled well was then placed in the geometric center of the UV Crosslinker and treated for the appropriate time (0–120 s). This process was repeated for each time point using the remaining wells in the 12-well plate. Following treatment, the entire contents from each well were transferred to a microfuge tube, mixed with 600 µL Potato Dextrose Broth (BD Difco, Becton, NJ, USA), and incubated at ambient temperature (approximately 20°C) in a shaking incubator (200 rpm) for 6 h. Following incubation, samples were either analyzed immediately or stored at 4°C for up to 24 h prior to analysis. For germination assessment, two 10 µL aliquots were transferred from each sample to a glass microscope slide and visualized using a light microscope at 40× (AmScope M150 Series, Irvine, California). For each aliquot, 100 conidia

were counted as germinated or non-germinated. The percentage of germinated conidia was calculated using the average of the two technical replicates. Germination was defined by the visible presence of a germ tube. Germination assessments following UV-C treatment were replicated on 3 days using biologically independent inocula (*n* = 3). Data were analyzed using one-way analysis of variance (ANOVA) with Tukey’s HSD *post hoc* comparisons.

### Statistical analysis across methodology

All statistical analyses were performed using JMP Pro 18 (SAS Institute, Cary, NC, USA) with significance defined as *P* < 0.05. In addition to the method-specific analysis described in previous sections, a fixed effects mixed model was applied to evaluate overall impact of UV-C dose, inoculum type, and methodology on log reduction across antifungal assessment methods. Cross-effects between inoculum type and method were further examined using least squares analysis with Tukey’s *post hoc* comparisons.

## RESULTS

### Viable CFU counts of UV-C treated conidia and HFs

#### Agar surface treatment

**Fig 1:**
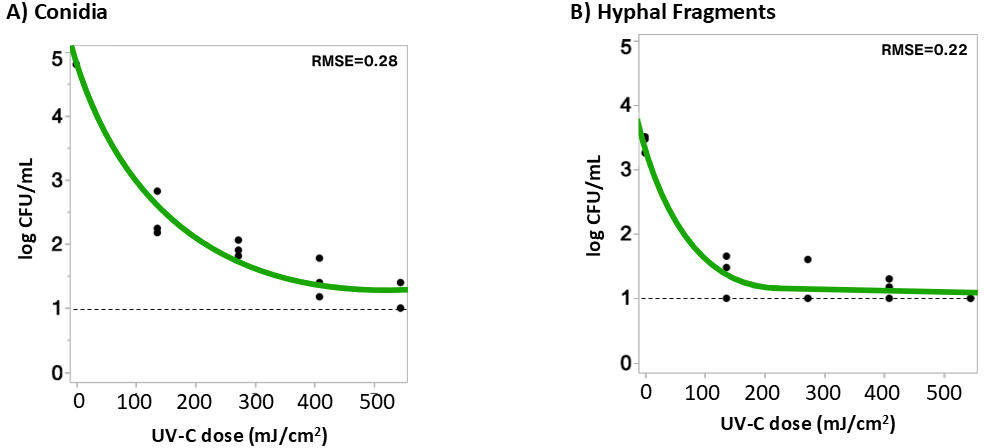
Viable *B. cinerea* BC01 (**A**) HF and (**B**) conidia following UV-C treatment on agar surfaces. *B. cinerea* were exposed to doses of UV-C (0–543.6 mJ/cm²) on PDA. Surviving populations were transformed to log CFU/mL. Each data point represents the result from an independent biological replicate for each treatment dose (*n* = 3). The dashed line indicates the limit of detection of the enumeration method. The green line indicates the mechanistic growth model fit for each data set with the RMSE displayed in the upper right-hand corner.

To evaluate UV-C efficacy on solid surfaces, *B. cinerea* conidia and HF were exposed to a range of doses, and viability was assessed by colony formation. The resulting data were modeled to describe inactivation kinetics of conidia (Fig. 1A) and HF (Fig. 1B). Overall, UV-C demonstrated non-linear, dose-dependent reductions for both inoculum types. A mechanistic growth model was fit to the observed survival data, yielding high model fidelity for both conidia (*R*^2^ = 0.966, root mean square error [RMSE] = 0.281) and HF (*R*^2^ = 0.952, RMSE = 0.224). The survival curves were described by the following equations for conidia:


$$y=1.121\times (1+3.040e^{-0.735x})$$

and for HF:


$$y=1.100\times (1+2.099e^{-1.514x})$$

where *x* is the UV-C dose (mJ/cm^2^) and *y* is the surviving fungal cell population (log CFU/mL). At the lowest treatment dose (135.9 mJ/cm^2^), conidia count decreased by 2.50 ± 0.15 log compared to untreated control, while the highest dose (543.6 mJ/cm^2^) resulted in 3.79 ± 0.02 log reduction, with two replicates reaching the limit of detection. HF showed slightly lower reductions of 2.03 ± 0.43 log and 2.41 ± 0.13 log at the same respective doses. The dose-response curves illustrate the susceptibility of both fungal structures towards UV-C and demonstrate a diminishing return in efficacy at higher doses. While increased UV-C exposure resulted in greater CFU reduction, the rate of inactivation plateaued at higher doses, with HF inactivation curve reaching plateau more rapidly due to reaching the limit of detection earlier (7 out of the 15 total replicates). Statistical analysis using a fixed effects mixed model with Tukey’s HSD *post hoc* tests showed that conidia and HF responded differently to UV-C treatment. However, tailing effects and variation in starting densities warrant cautious interpretation when comparing reductions at a given treatment.

#### Liquid suspension treatment

**Fig 2:**
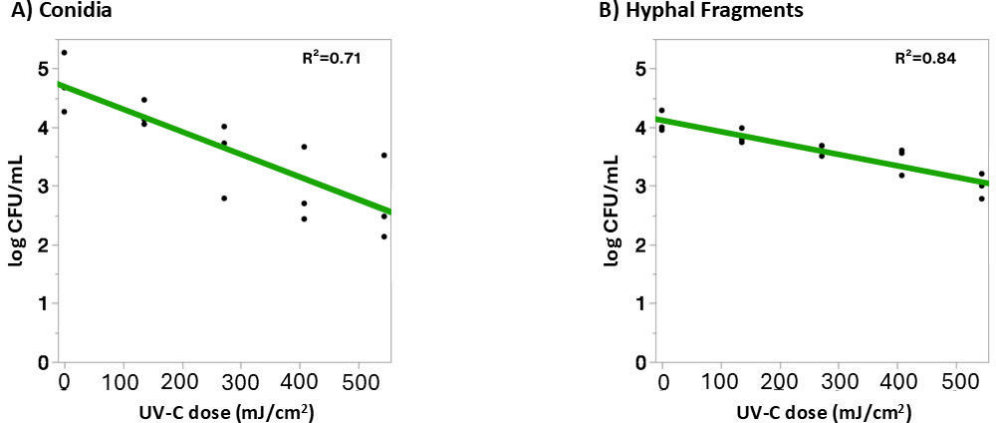
Viable *B. cinerea* BC01 (**A**) conidia and (**B**) HFs following UV-C treatment in liquid suspension. *B. cinerea* were exposed to doses of UV-C (0–543.6 mJ/cm²) in DI water. Surviving populations were transformed to log CFU/mL. Each data point represents the result from an independent biological replicate for each treatment dose (*n* = 3). The green line indicates the linear model fit for each data set with the *R*^2^ displayed in the upper right-hand corner.

Liquid *B. cinerea* conidia and HF suspensions were exposed to the same chosen range of UV-C doses with surviving populations assessed by colony counts (Fig. 2). A linear model was fit to the observed survival data, yielding moderate model fidelity for both conidia (*R*^2^ = 0.708, RMSE = 0.519) and HF (*R*^2^ = 0.837, RMSE = 0.171). The survival curves were described by the following equations for conidia:


y=4.68+−0.39x

and for HF:


y=4.11+−0.19x

Similarly, in this model, *x* is the UV-C dose (mJ/cm^2^), and *y* is the surviving fungal cell population (log CFU/mL). Both conidia and HF exhibited dose-dependent reductions in viability (generalized linear mixed model, HF: *P* = 0.0011, conidia: *P* = 0.0067). At the highest UV-C dose (543.6 mJ/cm^2^), conidia showed an average reduction of 2.02 ± 0.82 log CFU/mL, while HF achieved 1.08 ± 0.39 log reduction. At intermediate doses (135.9, 271.8, and 407.7 mJ/cm^2^), log reductions for HF (0.24 ± 0.74, 0.45 ± 0.17, and 0.63 ± 0.22 log, respectively) were also lower than conidia (0.52 ± 0.30, 1.23 ± 0.49, 1.80 ± 0.78 log, respectively). A Tukey’s *post hoc* analysis of a fixed effect mixed model grouped conidia and HF separately, showing statistically different responses to UV-C treatment (*P* < 0.0001). HF (Fig. 2B) exhibited a shallower linear dose–response slope than conidia (Fig. 2A), indicating comparatively lower sensitivity to UV-C.

### Fungal growth kinetics

**Fig 3:**
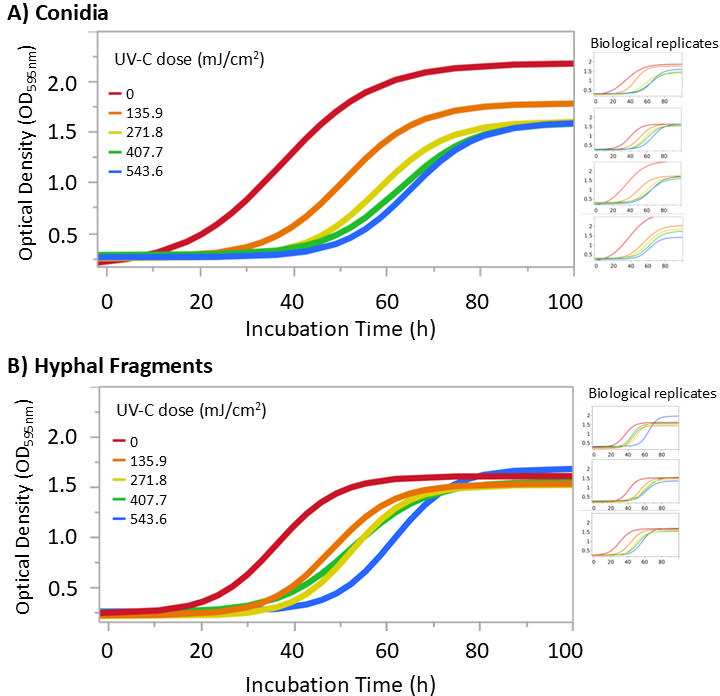
Growth kinetics of *B. cinerea* BC01 (**A**) conidia and (**B**) HFs following UV-C treatment of liquid fungal suspensions based on changes in OD (OD_₅₉₅_) of PDA (ambient temp, 96 h). Growth curves were fit using the 4P model (conidia: *n* = 4; HF: *n* = 3). Biological replicates are displayed on the right side of the main graph.

To evaluate effects of UV-C treatment on *B. cinerea* conidia and HF over extended incubation, we monitored fungal growth over 96 h using OD-based growth kinetics on PDA (Fig. 3A and B). OD values represent the accumulation of hyphal biomass, which can be a function of both the number of germinated units and length of elongated hyphae. As such, the resulting growth curves provide insight into the overall impact of post-UV-C treatment on fungal development. The key parameters investigated (Table 1) were the maximum growth rate (*G*_*R*_), the inflection point (*T*_IP_), and the △LPD. *G*_*R*_ represents the maximum slope of the 4P model and indicates the maximum growth rate (OD/h). *T*_IP_ corresponds to the time (h) at which this maximum growth rate is achieved. △LPD, calculated as the difference in *T*_IP_ between control and treated samples, reflects the delay in the onset of growth due to UV-C exposure (the change in LPD).

**TABLE 1:** Growth kinetics parameters of UV-C treated *B. cinerea* BC01 conidia and HFs based on 4P model fit of individual biological replicates displayed in Fig. 3^*c*^^,^^*d*^

Propagule type	UV-C dose (mJ/cm^2^)	Growth rate (OD_595_/h)	*T*_IP_ (h)	Change in LPD (ΔLPD) (h)^*a*^	Predicted cell density (log CFU/mL)^*b*^
Conidia	0.0	0.11 ± 0.03^a^	37.35 ± 5.15^a^		5.00 ± 0.15^a^
	135.9	0.14 ± 0.02^ab^	51.30 ± 4.94^b^	13.95 ± 4.59^a^	4.53 ± 0.18^b^
	271.8	0.14 ± 0.01^b^	58.20 ± 3.44^c^	20.85 ± 4.12^b^	4.26 ± 0.14^c^
	407.7	0.14 ± 0.02^ab^	63.19 ± 3.82^d^	25.85 ± 4.83^c^	4.04 ± 0.17^d^
	543.6	0.15 ± 0.03^b^	65.06 ± 3.99^d^	27.72 ± 4.13^c^	3.96 ± 0.18^d^
HF	0.0	0.18 ± 0.03^A^	35.96 ± 4.63^A^		2.74 ± 0.28^A^
	135.9	0.19 ± 0.04^A^	48.00 ± 5.35^B^	12.03 ± 2.66^A^	2.16 ± 0.20^B^
	271.8	0.19 ± 0.04^A^	52.59 ± 4.49^B^	16.63 ± 3.05^B^	1.99 ± 0.15^B^
	407.7	0.18 ± 0.04^A^	52.81 ± 7.51^B^	16.85 ± 6.95^B^	1.99 ± 0.25^B^
	543.6	0.20 ± 0.05^A^	61.09 ± 5.48^C^	25.13 ± 5.23^C^	1.72 ± 0.16^C^

^*a*^ Increase in LPD was calculated by subtracting the LPD of the untreated cell suspensions (0 mJ/cm^2^).

^*b*^ Predicted log CFU/mL was calculated using an inverse prediction of UV-C treated growth curve *T*_IP_ values from an exponential 3P decay model fitting the known log CFU/mL of diluted samples to observed LPD (see Table S1).

^*c*^ Values are expressed as the mean ± standard deviation of well replicates (conidia: *n* = 12; HFs: *n* = 16). Results that do not share superscript letters denote statistical differences in growth curve parameters between UV-C treatments within each inoculum type (HF: uppercase; conidia: lowercase) (*P* < 0.05, standard least squares analysis with Tukey’s HSD *post hoc* test).

^*d*^ The uppercase and lowercase letters distinguish statistical comparisons conducted separately for the two propagule types. Within each inoculum type (hyphal fragments vs. conidia), treatments that do not share a letter differ significantly (*P* < 0.05).

#### Impact of UV-C dose on growth dynamics

**Fig 4:**
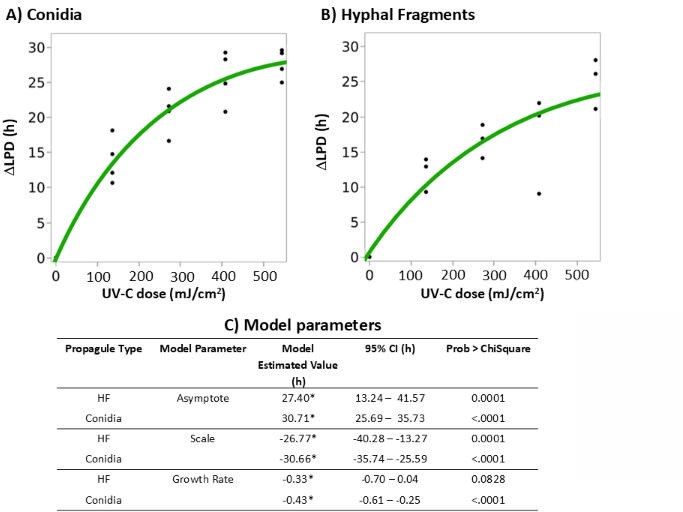
UV-C-induced changes in ΔLPD of *B. cinerea* BC01 conidia and HFs. (**A**) ΔLPD of conidia across UV-C doses, modeled using a 3P model. (**B**) ΔLPD of HF under identical conditions, fit to the same model. (**C**) Summary table of fitted model parameters for conidia and HF. Asterisks indicate parameter estimates with overlapping 95% confidence intervals.

Fungal growth kinetics from the untreated cell suspensions followed a typical sigmoidal pattern: (i) a lag phase with a stable OD reflecting the period where conidia have not yet germinated and HF are not yet elongating, (ii) an exponential phase of OD increase indicating hyphae proliferation, and (iii) a post-exponential phase where OD has reached a maximum value (Fig. 3). Generally, UV-C treatment caused a dose-dependent shift of the growth curves with an extended LPD, calculated in relation to the *T*_IP_ for both conidia and hyphae inoculum compared to controls, indicating a delay in biomass formation. Specifically, *T*_IP_ was significantly extended in all UV-C treated conidia and HF suspensions compared with untreated controls (*P* < 0.05), with higher UV-C doses correlated with longer lag phases. Increases in LPD (△LPD) allowed for direct comparison between conidia and HF regardless of differences in initial CFU density (Fig. 4). Notably, although △LPD values for conidia were always higher than HF, the two were not significantly different as indicated by overlapping 95% confidence intervals at equivalent UV-C doses (Fig. 4C). This suggests a similar delay in growth onset in response to UV-C, regardless of the types of fungal propagule.

### Conidial germination assessment

**Fig 5:**
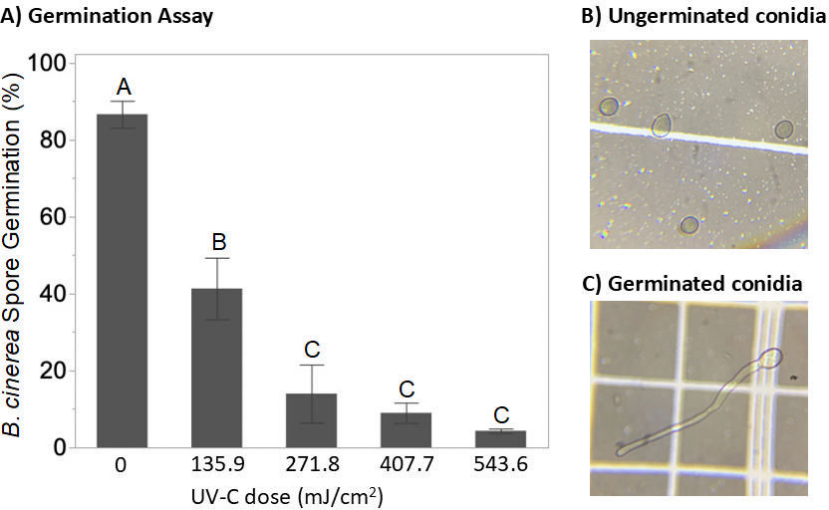
(**A**) *B. cinerea* BC01 conidia germination following UV-C treatment (0–543.6 mJ/cm^2^). Bars represent the mean germination rate (%) at each UV-C dose in independent replicates (*n* = 3). Error bars denote standard deviation. Treatments not sharing the same capital letter are significantly different (*P* < 0.05, one-way ANOVA with Tukey’s HSD *post hoc* test). (**B**) Light microscopy image (40×) of *B. cinerea* conidia. (**C**) Germinated *B. cinerea* conidium displaying apical extension of the germ tube and developing hyphae.

Figure 5A shows the conidial germination rate as a function of UV-C dose, with representative images of ungerminated and germinated conidia in Fig. 5B and C, respectively. In untreated suspensions, ~87% of conidia germinated, whereas UV-C exposure significantly reduced germination in a dose-dependent manner. Germination rates were reduced to 41.33 ± 8.02% at the lowest dose (135.9 mJ/cm^2^), and 4.33 ± 0.58% at the highest dose (543.6 mJ/cm^2^). Germination rates plateaued at higher UV-C doses with no statistically significant difference observed across the three highest doses tested (i.e., 271.8, 407.7, and 543.6 mJ/cm²; *P* > 0.05, Tukey’s HSD).

### Comparison across different assessment methods for antifungal efficacy

**Fig 6:**
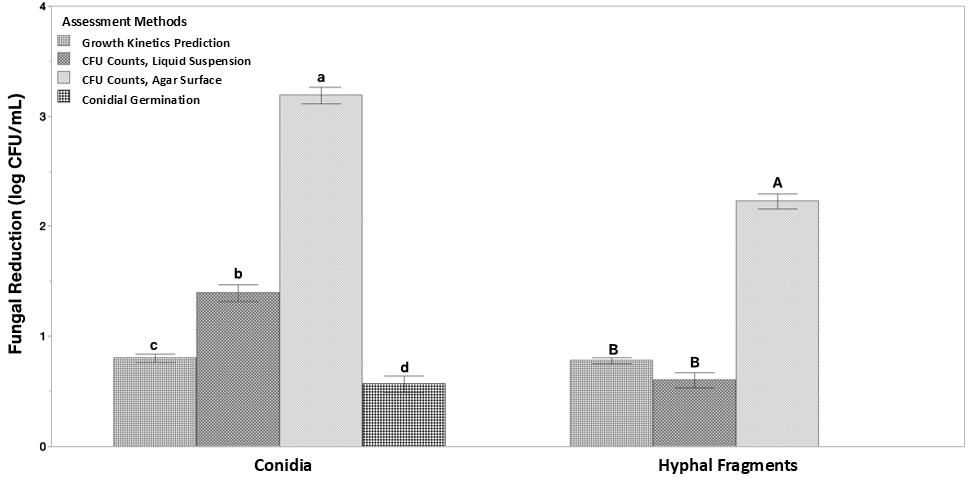
Reduction of *B. cinerea* BC01 in response to UV-C treatment as a function of assessment methodology and inoculum type. Bars represent mean reductions (log CFU/mL) across all UV-C doses grouped by assessment methodology. Error bars represent model standard error (conidia *n* = 77, HF *n* = 91). Within each inoculum type, bars not sharing the same letter (HF: uppercase; conidia: lowercase) are significantly different (*P* < 0.05, fixed-effects mixed model with Tukey’s HSD *post hoc* test).

A mixed-effects model analysis of all log reduction data confirmed that UV-C dose, propagule type, and assessment method (dose × inoculum × method) each significantly affected the observed *B. cinerea* viability (*P* < 0.0001). Tukey’s *post hoc* analysis further revealed that agar surface treatment consistently yielded significantly (*P* < 0.05) higher log reductions at each treatment dose compared to other assessment methods (Fig. 6). It was observed that germination assays produced the lowest predicted log reductions. Notably, observed germination rates were significantly higher than those predicted based on CFU-derived estimates (Table 2), particularly at moderate and high UV-C doses. For example, at 543.6 mJ/cm², germination assays indicated about 4% conidia viability, whereas agar surface CFU counts reflected about 99.98% reduction or approximately 0.02% conidia viability.

**TABLE 2:** Comparison of UV-C treatment effects on *B. cinerea* BC01 conidia, expressed as percent viability based on viable CFU counts (agar surface and liquid suspension) and conidial germination assay^*b*^

UV-C dose (mJ/cm²)	Agar surface	Suspension	Conidial germination
0.0	100.00 ± N/A%^*c*^	100.00 ± N/A%	100.00 ± N/A%^*a*^
135.9	0.31 ± 0.10%^A^	30.26 ± 38.43%^B^	41.33 ± 8.02%^B^
271.8	0.10 ± 0.02%^A^	5.93 ± 11.14%^B^	14.00 ± 7.55%^C^
407.7	0.03 ± 0.01%^A^	1.58 ± 2.99%^B^	9.00 ± 2.65%^C^
543.6	0.02 ± 0.00%^A^	0.37 ± 0.48%^B^	4.33 ± 0.58%^C^

^*a*^ Conidial germination at 0 mJ/cm² was measured as 86.67 ± 3.51%, and this value was normalized to 100% for comparative purposes. Therefore, 86.67 ± 3.51% is considered to represent 100% viable cells.

^*b*^ Values represent mean ± standard deviation (*n* = 3 biological replicates). A mixed model on these values (log transformed) with a Tukey’s *post hoc* was performed. Values not sharing the asterisk symbols are statistically different (*P* < 0.05). Uppercase letters indicate values included in the statistical analysis (across UV-C doses).

^*c*^ “N/A” indicates that the value is not an observed measurement but the reference point to which all other germination percentages were scaled.

## DISCUSSION

This study systematically evaluated antifungal efficacy of UV-C against *B. cinerea* conidia and HF using multiple *in vitro* assessment methods: viable CFU counts from agar surface treatment and liquid suspension treatment, OD-based growth kinetics, and conidial germination assay. Across all assays, UV-C treatment resulted in significant, dose-dependent reductions in viability of both inoculum types; however, the perceived magnitude of its efficacy varied significantly with the method of assessment. These differences underscore that while UV-C is broadly effective, its apparent efficacy is influenced by the experimental context. This finding is important as much of the existing UV-C literature relies on a single endpoint (often conidial germination), which may overestimate viability compared to other methods. Direct comparison of antifungal assessment methods in this study demonstrates that the choice of assay influences interpretation of treatment outcomes and carries important implications for the development of industry-relevant UV-C interventions.

A particularly novel aspect of this study was the parallel evaluation of HF alongside conidia. Unlike bacteria, filamentous fungi such as *B. cinerea* can reproduce through both conidia and HF, each serving as a viable infectious unit (26). Fragmentation is often overlooked in fungal studies despite their relevance in postharvest environments, where mechanical damage and close crop contact facilitate hyphal fragmentation and dispersal. Our results show that HF tended to be more resistant to UV-C than conidia, suggesting that HFs may pose a greater challenge for sanitation in storage and packing facilities. As most prior studies have focused primarily on conidial (17, 27, 28), these findings expand current understanding and highlight the importance of including HF in both research and practical applications when evaluating UV-C efficacy for postharvest control.

### Method-based interpretations of UV-C efficacy

CFU counts provide a direct measure of viability based on the ability of individual cells to recover and proliferate into colonies over a 72-h period. In this study, UV-C treatment led to substantial reductions in both conidia counts and HF counts, particularly in the surface-based assay. This finding aligns with previous results with higher reductions of conidia of *Aspergillus flavus*, *Aspergillus niger*, *Penicillium corylophilum*, and *Eurotium rubrum* on UV-C exposed agar surfaces compared to UV-C treated liquid suspensions (29). On agar surfaces, conidia are located at or near the surface and thus receive more uniform and direct exposure to UV-C. In contrast, in liquid suspensions, conidia are distributed throughout the volume, and those in the deeper suspension may receive a reduced dose due to absorption and scattering effects. Even with the shallow suspension depth used in this study (~1.2 mm), uniform dose delivery could theoretically still be compromised. These findings highlight that the physical format of UV-C application (e.g., surface vs suspension) significantly impacts its antifungal efficacy. These findings of log-linear UV-C reduction are consistent with observations of bacteria in liquid food products (30) and in *Phytophthora* sp. zoospores in water (31), suggesting that suspension-based systems may share similar patterns of UV-C inactivation dynamics across different microorganisms. Finally, temperature monitoring during UV treatment showed only minor increases across all samples (ΔT ≤ 0.9°C; Table S2), indicating that heat effects did not contribute meaningfully to antifungal efficacy.

Conidial germination assays are useful for rapid analysis and capturing early-stage fungal responses to UV-C treatment. HFs are excluded from this analysis. While commonly used, this method does not account for the viability of mycelial outgrowth or conidia that may not germinate readily (15). We used a 6-h incubation time, a typical time for many fungal conidia including *B. cinerea* to initiate germination. However, this fixed duration may have excluded conidia that were viable but exhibited delayed germination due to natural heterogeneity or sublethal damage from UV-C exposure. Moreover, this assay is typically limited in the ability to determine the true “viability.” Specifically, we hypothesize that conidia that initiate germination but are unable to sustain hyphal elongation or colony formation, due to suboptimal environmental conditions or genetic damage, would still be counted as viable using this assay; thus, there is a tendency for the conidial germination assay to overestimate viability. The conidial germination assay did result in the highest viable cell density compared to all other methods. For instance, at 543.6 mJ/cm², germination was still observed in ~4% of conidia, whereas CFU counts from surface plating showed >99.98% reduction in viable conidia (Table 2). The use of differential dyes, like Trypan blue, may help differentiate viability but may introduce cytotoxic artifacts. Despite these constraints, germination data in this study displayed a clear dose-dependent decline with diminishing efficacy at higher doses, which was consistent with results from both viable CFU counts and OD-based growth curves.

Growth kinetics on solid agar of UV-C treated conidia and HF suspensions were used to quantify biomass accumulation throughout a 96-h incubation. Solid agar was selected to mimic surface colonization by *B. cinerea*. For both conidia and HF, UV-C treatment led to a dose-dependent delay in growth, as reflected by extended LPD as rightward shifts in *T*_IP_ (Fig. 3 and 4). While growth curves capture overall treatment effects, they do not inherently reflect whether growth delays are due to a reduction in viable cell number or from impaired proliferation of sublethally damaged cells. To explore this, *T*_IP_ values from UV-C-treated samples were compared with those from serially diluted, untreated cell suspensions of known cell density (Table S1). This enabled the construction of a predictive model correlating *T*_IP_ to initial cell density, allowing prediction of viable cell density from UV-C-treated samples. These predicted cell densities, displayed in Table 1, were then compared to viable CFU counts from the same UV-C-treated suspension. If the predicted reductions closely match those obtained from plate counts, it would support the assumption that UV-C-induced growth delays (shift of *T*_IP_) result primarily from reduced viable cell numbers due to UV-C exposure. Conversely, significant discrepancies would suggest that sublethal metabolic impairment may also contribute to the observed growth delays. As shown in Fig. 6, for HF, predicted cell densities were similar to (*P* > 0.05) observed CFU counts from suspension treatment, suggesting that growth delays in HF primarily reflected reduced viable cell numbers. However, for conidia, predicted reductions were consistently lower than those observed by CFU counts in suspension. This discrepancy indicates that UV-C may induce sublethal physiological damage in some conidia that slows further development such as apical growth and germ tube extension, even after germination has occurred. As a result, conidia that fail to form colonies within the viable CFU count timeframe (72 h) may still contribute to biomass measurements during extended OD-based monitoring (96 h), potentially leading to overestimation of viability in growth kinetics assays. In addition, when comparing germination assay with viable CFU counts (Table 2), microscopically observed conidial germination rates were substantially higher than those predicted from viable CFU counts for both agar and suspension treatment. This further implies that UV-C may impair the developmental progression of surviving conidia into visible colonies.

Together, these findings emphasize the importance of methodological selection and the need for context-aware antifungal assessments. Employing a multi-method approach would enhance the understanding of biological impact of the treatment and guide the development of application-specific strategies. However, implementing multi-method approaches may not always be feasible in routine or industrial settings. Given this, germination assay and growth kinetics may serve as practical options and offer conservative estimates of treatment efficacy, particularly in liquid systems. Conversely, agar surface treatments, while potentially yielding higher inactivation estimates, may better reflect applications involving uniform solid surfaces or contact-based contamination. For industry adoption, careful dose optimization is also essential to balance effective fungal inactivation with energy efficiency, as the dose-effectiveness relationship is not always a linear relationship.

### Differential sensitivity of conidia and HFs

**Fig 7:**
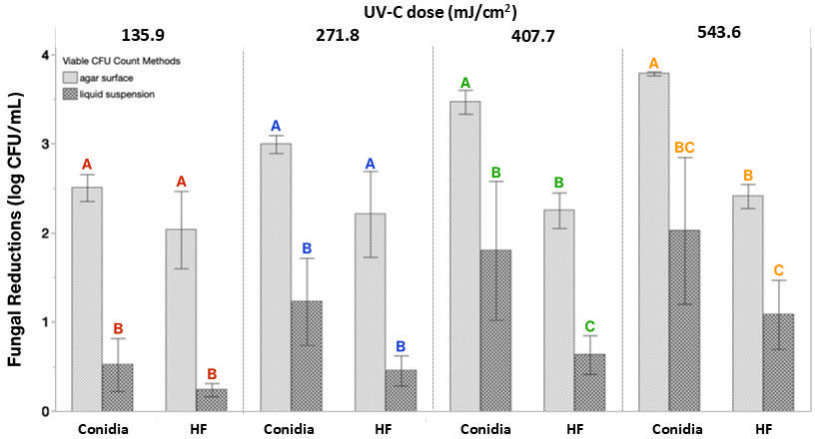
Differential sensitivity of *B. cinerea* BC01 conidia and HFs to UV-C treatments (135.9–543.6 mJ/cm^2^) between liquid suspension and agar surface treatment methods. Reductions (log CFU/mL) were compared between inoculum types using viable CFU count data following surface and suspension treatments. A fixed-effects least squares model evaluated the interaction between inoculum type and method at each UV-C dose (mJ/cm²), with significance determined by Tukey’s HSD *post hoc* test (*P* < 0.05). Colored letters indicate data sets that were used for statistical analysis (i.e., by UV-C dose). Bars represent mean log reductions (*n* = 3 biological replicates). Error bars display standard deviations.

In this study, HF showed generally lower log reductions than conidia at equivalent UV-C doses. Although not always statistically significant, the trend suggests that HF may possess greater resistance to UV-C (Fig. 7). Resistance to UV irradiation can be attributed to multiple factors including cell wall thickness, protective pigment content, and reducing surface area of exposure. Interestingly, previous studies reported that *B. cinerea* conidia have thinner cell walls (0.5 µm (32) than hyphae (0.1–0.2 µm (33), which contradicts a cell wall thickness-based explanation for the greater UV susceptibility of conidia. Instead, differences in cell wall composition may offer a more plausible explanation. Hyphae and conidia contain melanin in their cell walls (34), a pigment known to protect fungi from UV-induced damage. Chitin, another structural component of the fungal cell wall, is also capable of absorbing UV wavelengths. The concentration of chitin tends to increase as hyphae mature (35, 36), potentially contributing to UV resilience as well. Differences in the level of these UV-resistant components may account for some protection against UV-C. An additional consideration is the surface area available for UV-C exposure. Conidia, due to their spherical shape and tendency to remain well-dispersed in suspension, likely present a larger cumulative surface area for uniform UV-C penetration. In contrast, HFs, which are often irregular in shape and prone to clustering, may experience partial shielding as overlapping structures reduce direct light exposure. Further research is needed to characterize the structural and biochemical properties that influence UV susceptibility in different fungal forms.

### Conclusion

This study provides a comprehensive *in vitro* evaluation of UV-C treatment efficacy against *B. cinerea* conidia and HF using a multi-method approach. UV-C exposure significantly reduced fungal viability in a dose-dependent manner across all assays, though the extent of efficacy varied by propagule type, treatment matrix, and assessment method. Under the same treatment conditions, surface-based treatments achieved the highest reductions, whereas lower efficacy in suspension-based treatments was likely due to physical constraints such as light scattering and shielding effects. Germination assays tended to overestimate viability, likely due to the inclusion of conidia that initiated germination but could not sustain hyphal elongation or colony formation. Growth kinetics revealed dose-dependent delays in biomass development, providing additional insight into sublethal impacts of UV-C on fungal propagules. Importantly, conidia and HF exhibited different responses to UV-C, with conidia generally showing greater sensitivity. Taken together, this study adds to the current body of knowledge by showing that both the fungal propagule type and the assessment method used can significantly influence measured UV-C efficacy, which are realistic and industry-relevant factors when evaluating UV-C as a postharvest sanitation tool for *B. cinerea* management. Future research should focus on validating these findings under commercial settings, optimizing UV-C dose to balance antimicrobial efficacy and energy efficiency, assaying pathogenicity to confirm whether UV-C-treated conidia and HF retain the ability to infect host tissue, and developing practical strategies to enhance treatment performance on complex matrices.

## Data Availability

The data generated in this study are available in the main text, presented figures, tables, and supplemental material. Data requests should be addressed to qingyang.wang@oregonstate.edu.

## SUPPLEMENTAL MATERIAL

Graphical abstract aem.01413-25-s0001.tif Visual depiction of the study.

Supplemental tables aem.01413-25-s0002.docx Tables S1 and S2.

ASM does not own the copyrights to Supplemental Material that may be linked to, or accessed through, an article. The authors have granted ASM a non-exclusive, world-wide license to publish the Supplemental Material files. Please contact the corresponding author directly for reuse.
