# Supplementary figures and images for: Multi-method in vitro assessment of ultraviolet-C treatment against conidia and hyphal fragments of Botrytis cinerea
Source: Appl Environ Microbiol. 2025 Nov 24;91(12):e01413-25. doi: 10.1128/aem.01413-25 (PMC12724258; doi:10.1128/aem.01413-25)

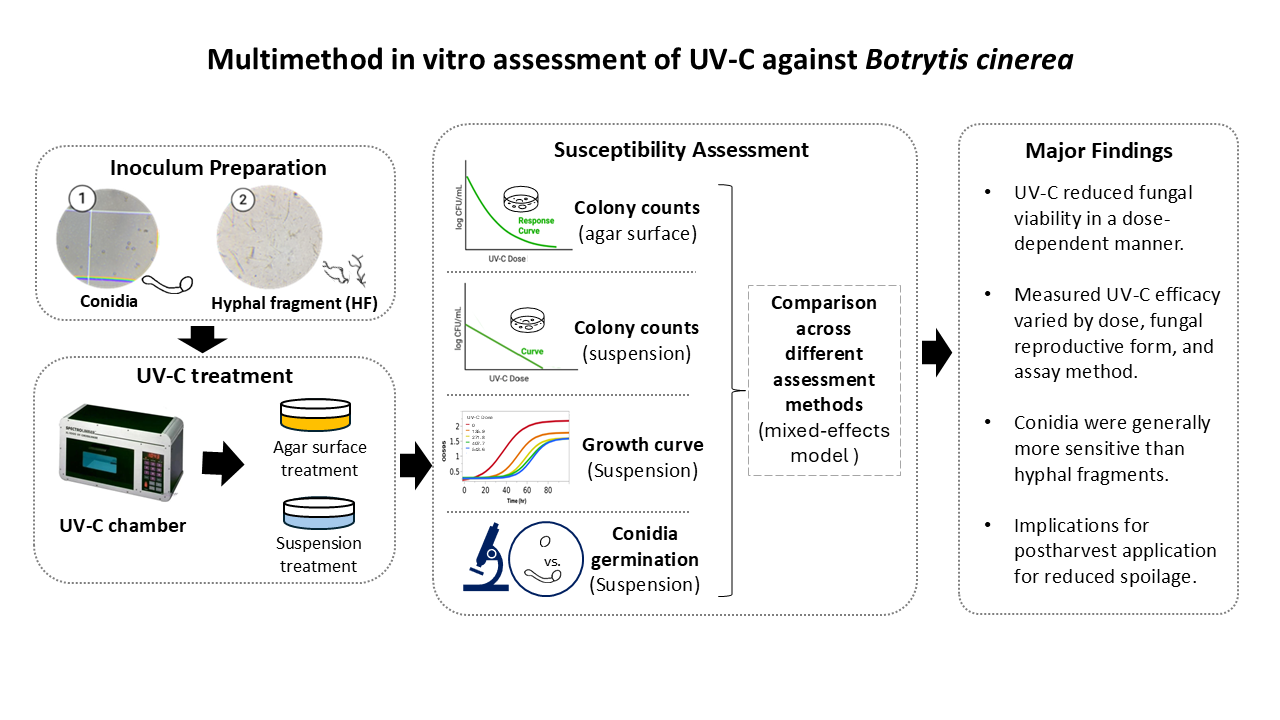

Supplement: Graphical abstract — Visual depiction of the study. [file aem.01413-25-s0001.tif]
