# Supplementary material for: Multi-method in vitro assessment of ultraviolet-C treatment against conidia and hyphal fragments of Botrytis cinerea
Source: Appl Environ Microbiol. 2025 Nov 24;91(12):e01413-25. doi: 10.1128/aem.01413-25 (PMC12724258; doi:10.1128/aem.01413-25)
Supplement: Supplemental tables — Tables S1 and S2. [file aem.01413-25-s0002.docx]

**Supplemental Materials**

**Multimethod in vitro assessment of ultraviolet-C treatment against conidia and hyphal fragments of *Botrytis cinerea***

Makayla Bellino^1^, Joy Waite-Cusic^1^, Qingyang Wang^1*^

^1^Department of Food Science and Technology, Oregon State University, Corvallis, OR, 97331

*Corresponding author

Qingyang Wang, Ph.D.

Department of Food Science and Technology

Oregon State University,

100 Wiegand Hall, Corvallis, OR 97331

Phone: 541-737-7611

Email: [qingyang.wang@oregonstate.edu](mailto:qingyang.wang@oregonstate.edu)

**Supplementary Table 1**: Growth kinetics parameters of *Botrytis cinerea* BC01 conidia and hyphal fragments suspension dilutions based on logistic 4-parameter model fit of individual biological replicates. These values were used for predicting viable cell density to support comparisons across assessment methods. Values represent mean ± standard deviation (n = 3 biological replicates^1^).

| Propagule Type | Dilution | Inoculum Density  (Log CFU/mL) | Growth Rate (OD_595nm_/h) | Time to Inflection Point (T_IP_) (h) | Change in Lag Phase Duration (ΔLPD) (h)^2^ |
| --- | --- | --- | --- | --- | --- |
| Conidia | Undiluted | 4.96 | 0.08 ± 0.01 | 38.71 ± 5.65 |  |
|  | 10^-1^ | 3.96 | 0.08 ± 0.00 | 65.10 ± 7.44 | 26.39 ± 7.44 |
|  | 10^-2^ | 2.96 | 0.08 ± N/A | 82.45 ± N/A | 43.74 ± N/A |
| Hyphal Fragments | Undiluted | 3.40 | 0.16 ± 0.02 | 26.68 ± 0.99 |  |
|  | 10^-1^ | 2.40 | 0.16 ± 0.02 | 42.05 ± 3.21 | 15.37 ± 3.21 |
|  | 10^-2^ | 1.40 | 0.13 ± 0.02 | 73.54 ± 5.32 | 46.86 ± 5.32 |

^1^For conidia suspensions at 10^-2^ dilution, n=1 due to insufficient growth for reliable curve fitting.

^2^Increase in lag phase duration was calculated by subtracting the lag phase duration of the untreated cell suspensions (0 mJ/cm^2^).

**Supplementary Table 2:** Temperature change in samples treated with the highest UV-C dose (120 s, 543.6 mJ/cm²). Surface temperatures of agar and suspension samples were measured before and after treatment using an infrared thermometer at two random locations (n = 2). ΔT was calculated as the difference between the mean temperature at 0 s and 120 s.

| Sample | T_0_ (ºC) | | T_120s_ (ºC) | | ΔT (ºC) | |
| --- | --- | --- | --- | --- | --- | --- |
|  | Rep 1 | Rep 2 | Rep 1 | Rep 2 | Average |  |
| Agar surface (PDA) | 26.0 | 25.9 | 26.8 | 26.9 | 0.9 |  |
| DI water 400mL | 26.6 | 26.7 | 27.2 | 27.4 | 0.7 |  |
| Conidia suspension 400mL | 26.6 | 26.6 | 27.1 | 27.3 | 0.6 |  |
| HF suspension 400mL | 26.6 | 26.5 | 27.4 | 27.3 | 0.8 |  |
